# Tirzepatide Reduces Fat Mass and Provides Good Glycaemic Control in Type 2 Diabetes Patients Undergoing Haemodialysis: A Single‐Centre Retrospective Study

**DOI:** 10.1002/edm2.489

**Published:** 2024-05-08

**Authors:** Akira Mima, Yasuhiro Horii

**Affiliations:** ^1^ Department of Nephrology Osaka Medical and Pharmaceutical University Osaka Japan; ^2^ Seiwadai Clinic Nara Japan

**Keywords:** dry weight, hemodialysis, tirzepatide, type 2 diabetes

## Abstract

**Objective:**

Tirzepatide is an injectable peptide approved by the US Food and Drug Administration for the treatment of Type 2 diabetes (T2DM). Its weight‐loss effect primarily targets fat reduction; however, such effect on patients with chronic kidney disease (CKD) undergoing haemodialysis (HD) has not been reported.

**Methods:**

Nine patients with CKD undergoing HD received weekly tirzepatide doses (2.5–7.5 mg) once a week. Evaluations encompassed tirzepatide's impact on dry weight (DW) and body composition assessed at baseline and study conclusion using bioelectrical impedance analysis. This longitudinal study included nine patients, with a median age of 53 years and median HD duration of 4 years.

**Results:**

Tirzepatide treatment significantly decreased glycated albumin compared with the value at baseline (22.7 ± 5.4 vs. 18.3 ± 2.5%, *p* = 0.028, respectively). Significant reductions were observed in DW (−1.0 kg, *p* = 0.024) and body mass index (−0.6 kg/m^2^, *p* = 0.050) following tirzepatide administration. Total fat mass was also reduced, but not significantly (− 2.51% from baseline, *p* = 0.214). In contrast, skeletal muscle mass was not decreased (−1.02% from baseline, *p* = 0.722). No serious side effects other than nausea were observed during the study period.

**Conclusion:**

Tirzepatide effectively provides good glycaemic control in T2DM patients undergoing HD, decreasing DW by reducing body fat mass without increasing frailty risk.

## Graphical abstract

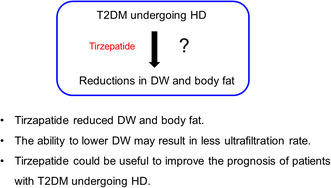

## 1 Introduction

The International Diabetes Foundation (IDF)'s report indicates a noteworthy rise in the prevalence of Type 2 diabetes (T2DM). Projections from this report suggest that approximately 783 million people globally could be affected by diabetes, with many patients being from African countries by 2035 [1]. Furthermore, studies have reported that T2DM is the seventh leading cause of mortality in the United States [2].

Diabetic kidney disease (DKD) is the main cause of chronic kidney disease (CKD). Despite numerous advances in the clinical management of DKD, its prevalence is rising steadily.

The increase in patients with DKD, demonstrating a high risk of progressing to end‐stage renal disease (ESRD) and necessitating renal replacement therapies such as haemodialysis (HD) [2]. Effective management of blood glucose is crucial in preventing DKD because the level of glucose control and onset of DKD are directly correlated [3, 4]. Additionally, the administration of renin–angiotensin–aldosterone (RAAS) inhibitors for controlling blood pressure appears to impart renoprotective effects [5, 6].

In T2DM, the intestine's L cells release incretin hormones, including glucagon‐like peptide (GLP‐1), which is pivotal in the regulation of blood glucose levels. GLP‐1 operates by increasing insulin secretion and decreasing glucagon secretion. In contrast, intestinal K cells secrete glucose‐dependent insulinotropic polypeptide (GIP) in response to food intake, stimulating insulin secretion to regulate blood glucose [7]. Recently, the GIP/GLP‐1 receptor agonist tirzepatide (Mounjaro, Lilly) has been approved for T2DM treatment by the US Food and Drug Administration. Tirzepatide induces a significant body weight loss, primarily by decreasing energy intake and enhancing energy expenditure in preclinical models, and has shown robust body weight reduction in patients with T2DM.

A combination of sodium‐glucose co‐transporter 2 inhibitors, incretin‐based therapies, renin–angiotensin system inhibitors and mineralocorticoid receptor blockers, namely the ‘DKD fantastic four’, has been proposed for managing DKD [8, 9]. However, the risk of ESRD persists despite the efficacy of these agents. Consequently, a significant optimism surrounds tirzepatide, a novel incretin‐based therapeutic agent, for its potential in treating DKD [10, 11].

To our knowledge, this is the first study to examine the efficacy and safety of tirzepatide as well as changes in body composition using bioimpedance analysis in patients with T2DM undergoing HD.

## 2 Material and Methods

### 2.1 Study Design and Collection of Medical Data

Patients who underwent stable outpatient HD at Seiwadai Clinic (Nara, Japan) for more than 6 months were retrospectively included in the study, whereas patients with insufficient data that affected the results and those with malignancy were excluded. Because the present study was conducted retrospectively by analysing data collected from routine clinical practice, the requirement for obtaining prior informed consent was waived by the Ethics Committee of Seiwadai Clinic. Finally, nine patients were included in the study. All procedures involving human participants were performed in accordance with the ethical standards of the National Research Committee and the 1964 Declaration of Helsinki and its later amendments or comparable ethical standards. The study was approved by the Ethics Committee of Seiwadai Clinic (approval no. 2023‐001). Baseline information for each participant, including age, duration of HD, duration of T2DM, medication, sex, blood pressure and dry weight (DW), was obtained from medical records. T2DM was identified on the basis of a diagnosis of diabetic retinopathy and/or a history of antidiabetic drug use.

Blood samples were obtained with the patients in supine position before and after HD at the beginning of the week. Bioelectrical impedance analysis (BIA) was performed in the supine position approximately 30 min after the HD session, when the DW set by the ultrafiltration rate was reached; intracellular water (ICW), extracellular water (ECW) and total body water (TBW), body fat content, skeletal muscle mass, protein content, bone mineral content, basal metabolism and skeletal muscle mass index (SMI) were measured with a body composition meter with multiple frequencies (1 kHz–1 MHz) using the ankle and wrist method [11, 12]. The InBody S10 (Biospace Co., Ltd., Seoul, Republic of Korea) was used for measurements. Cardiothoracic ratio was calculated on the basis of a chest radiograph. Body mass index (BMI) was calculated from DW and height: BMI = DW/height^2^ (kg/m^2^).

### 2.2 Data Analysis

Continuous variables are presented as median and mean values. Statistical significance was determined using the Wilcoxon test. All analyses were performed using StatView (SAS Institute) and Excel, and statistical significance was set at *p* < 0.05.

## 3 Results

Table 1 shows baseline characteristics of the nine patients (seven males and two females). The total observation period was 4 (interquartile range [IQR] 3–6) months, and the median age at onset was 53 (IQR 51–68) years. Furthermore, the median HD duration was 4 (IQR 3–5) years, and the median T2DM duration was 12 (IQR 11–20) years. Concomitant antidiabetic agents included insulin (70%), glinide (20%) and imeglimin (10%). Among the nine patients, six transitioned from dulaglutide to tirzepatide, whereas two switched from dipeptidyl peptidase‐4 (dpp‐4) inhibitors. Three patients eventually discontinued insulin. The average tirzepatide dosage administered was 4.7 ± 1.5 mg/week.

**TABLE 1:** Patient characteristics.

*n*	9
Male:female	7:2
Age (median; years)	53 (IQR: 51–68)
DW (median; kg)	70.5 (IQR: 68–80.5)
BMI (median; kg/m^2^)	24.3 (IQR: 23.3–27.7)
Duration of diabetes (median; years)	12 (IQR: 11–20)
Duration of HD (median; years)	4 (IQR: 3–5)
Observation period (median; months)	4 (IQR: 3–6)
Serum potassium (mEq/L)	4.8 ± 0.8
Total cholesterol (mg/dL)	154 ± 36
Triglyceride	176 ± 100
Concomitant antidiabetic medications (*n*)	10
Insulin (*n*)	7
Glinide (*n*)	2
Imeglimin (*n*)	1

Abbreviations: BMI, body mass index; DW, dry weight; HD, haemodialysis; IQR, interquartile range.

**FIGURE 1:**
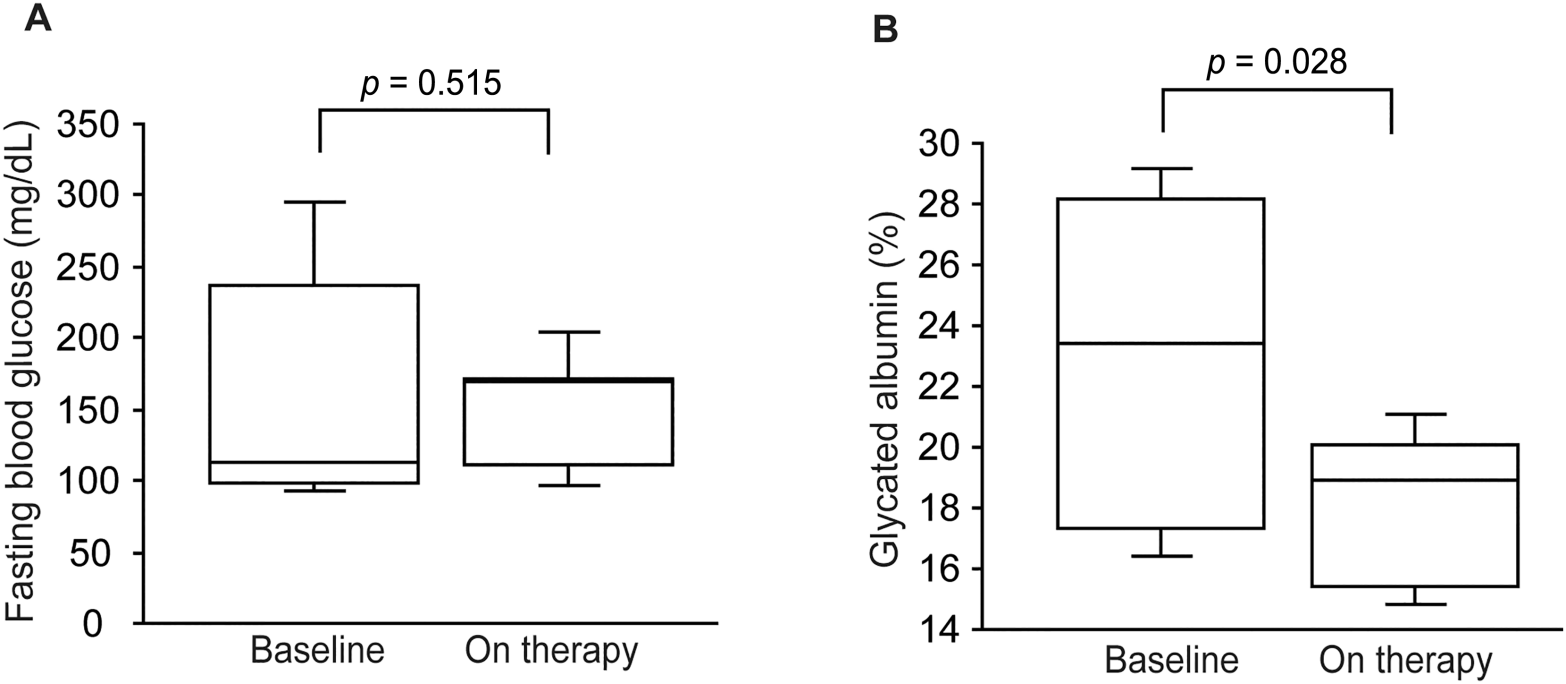
Box plots showing fasting blood glucose (A) and glycated albumin (B) at baseline and after tirzepatide treatment. The duration of tirzepatide treatment was 4 (IQR 3–6) months.

The baseline serum potassium, total cholesterol and triglyceride levels were 4.8 ± 0.8 mEq/L, 154 ± 36 mg/dL, and 176 ± 100 mg/dL, respectively. The baseline fasting blood glucose level was 167 ± 87 mg/dL, showing a decrease albeit not reaching statistical significance (151 ± 43 mg/dL, *p* = 0.515, Figure 1A). Tirzepatide treatment significantly decreased glycated albumin (GA) compared with baseline measurements (22.7 ± 5.4 vs. 18.3 ± 2.5%, *p* = 0.028, respectively, Figure 1B).

**FIGURE 2:**
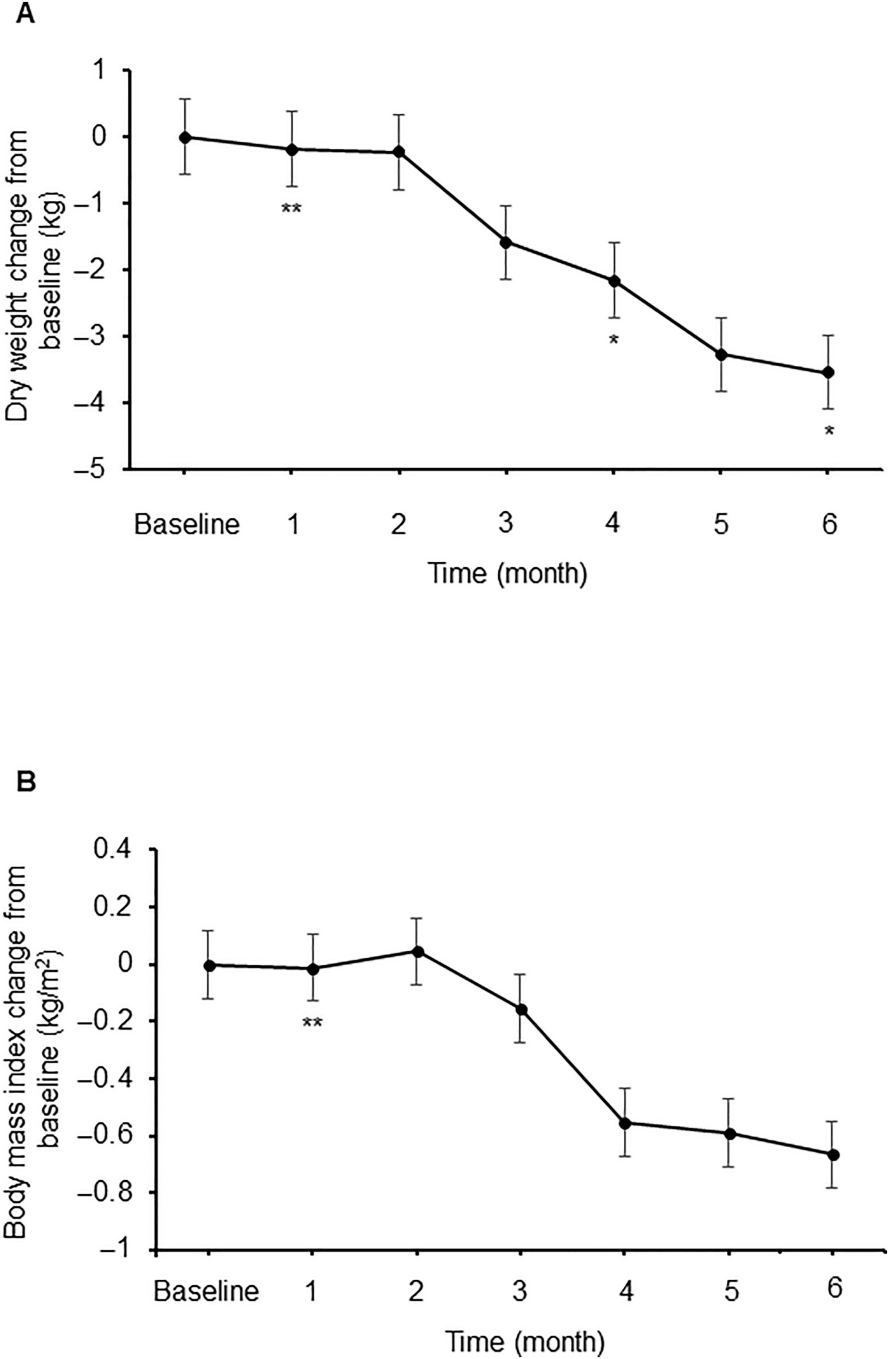
Change from baseline in dry weight (A) and body mass index (B). *p < 0.05 versus baseline, **p < 0.01 versus baseline.

**FIGURE 3:**
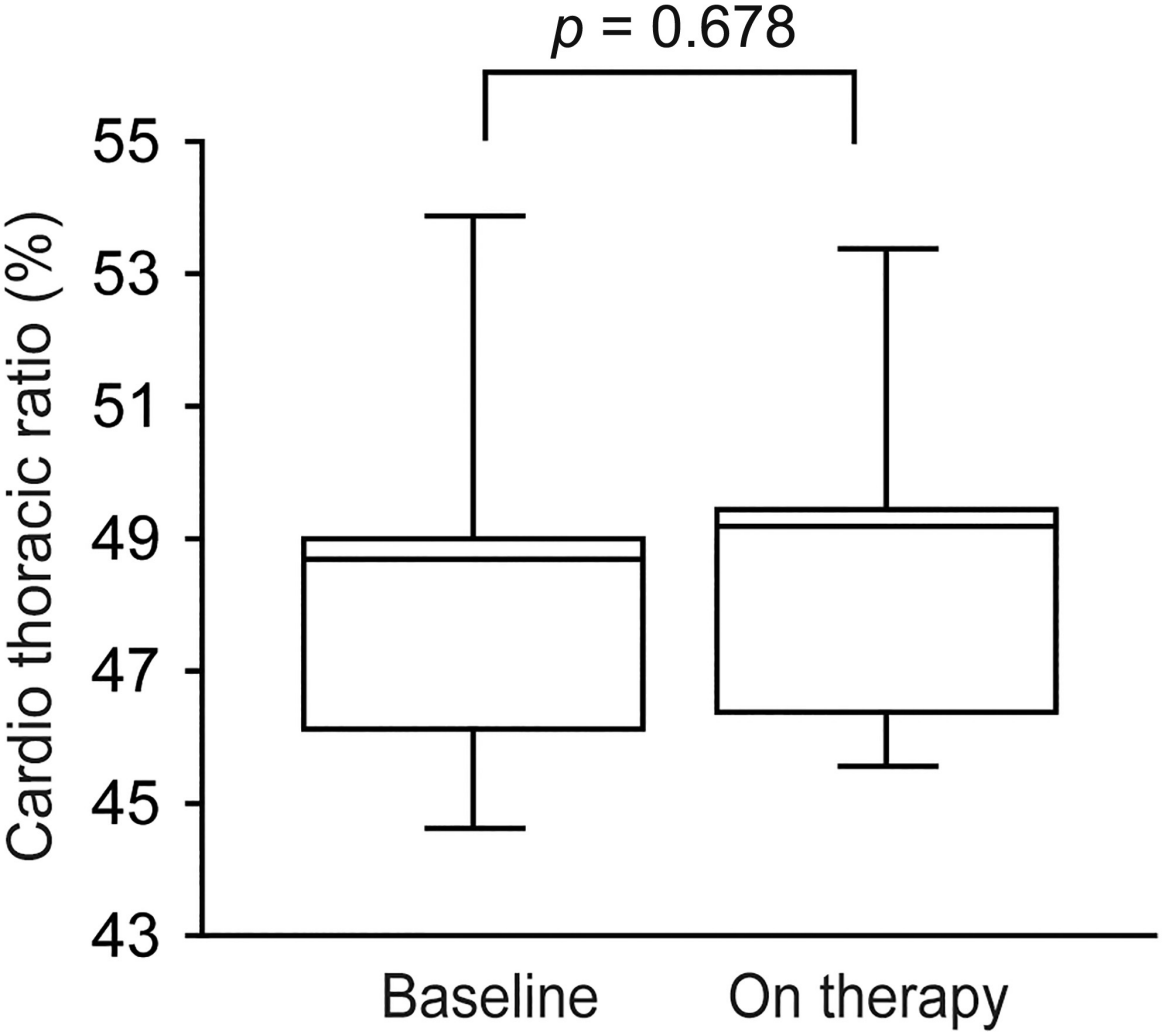
Box plots showing cardiothoracic ratio at baseline and after tirzepatide treatment.

DW and BMI at baseline were 70.5 kg (IQR 68–80.5) and 24.3 kg/m^2^ (IQR 23.3–27.7), respectively. In this cohort, tirzepatide administration resulted in statistically significant decreases in DW and BMI; specifically, DW decreased by −1.0 kg (IQR −2 to −0.5) and BMI by −0.6 kg/m^2^ (IQR −1.1 to 0.1) at the conclusion of the observation (*p* = 0.024 and *p* = 0.050, respectively) (Figure 2A,B). No change in CTR was observed before or after the administration of tirzepatide (48.5 ± 3.7 vs. 48.7 ± 3.2%, *p* = 0.678) (Figure 3).

**FIGURE 4:**
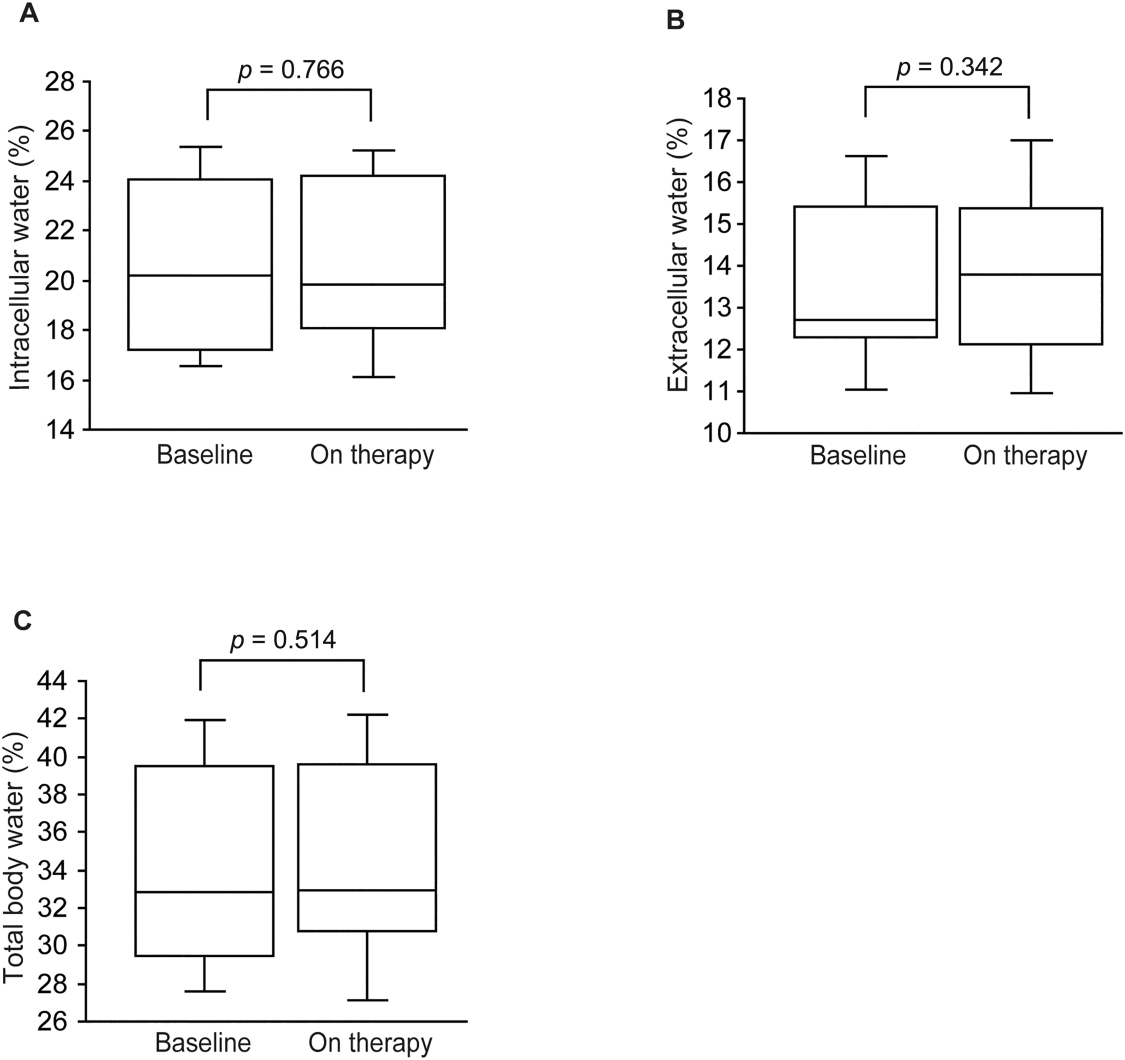
Box plots showing intracellular water (%) (A), extracellular water (%) (B) and total body water (%) (C) at baseline and after tirzepatide treatment. The duration of tirzepatide treatment was 4 (IQR 3–6) months.

Compared with pretreatment values, BIA data showed that ICW, ECW and TBW remained unchanged after tirzepatide administration (20.5 ± 3.6 vs. 20.6 ± 3.5%, *p* = 0.766; 13.6 ± 2.1 vs. 13.8 ± 2.2%, *p* = 0.342; 34.1 ± 5.7 vs. 34.4 ± 5.7%, *p* = 0.514, respectively) (Figure 4A–C).

**FIGURE 5:**
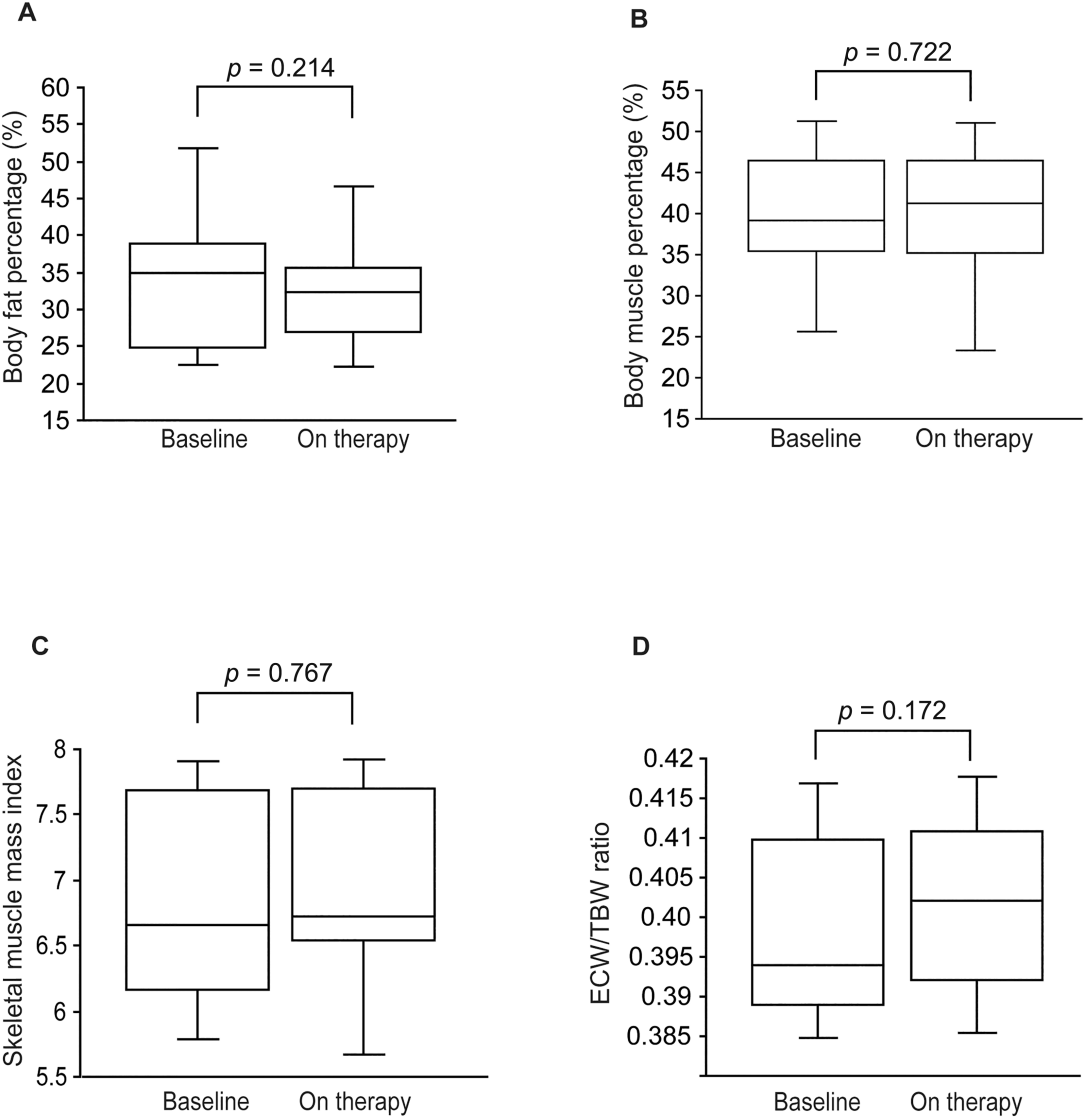
Box plots showing body fat (%) (A), body muscle (%) (B), skeletal muscle index (C) and extracellular water/total body water (D) at baseline and after tirzepatide treatment. The duration of tirzepatide treatment was 4 (IQR 3–6) months. ECW, extracellular water; TBW, total body water.

BIA data showed that the total fat mass was decreased after tirzepatide treatment, although not significantly (−2.51% from baseline; *p* = 0.214, Figure 5A). In contrast, skeletal mass and SMI that can be used to assess sarcopenia and frailty were not decreased by tirzepatide (−1.02% from baseline, *p* = 0.722; 1.17% from baseline, *p* = 0.767, respectively) (Figure 5B,C).

Given the suggested association of ECW/TBW ratio with protein‐energy wasting and the elevated mortality risk linked to a ratio surpassing 0.4 [13], our study delved into assessing this ratio within the study cohort. Induction of tirzepatide did not affect the ECW/TBW ratio (0.40 ± 0.01 vs. 0.40 ± 0.01; *p* = 0.172, Figure 5D).

**FIGURE 6:**
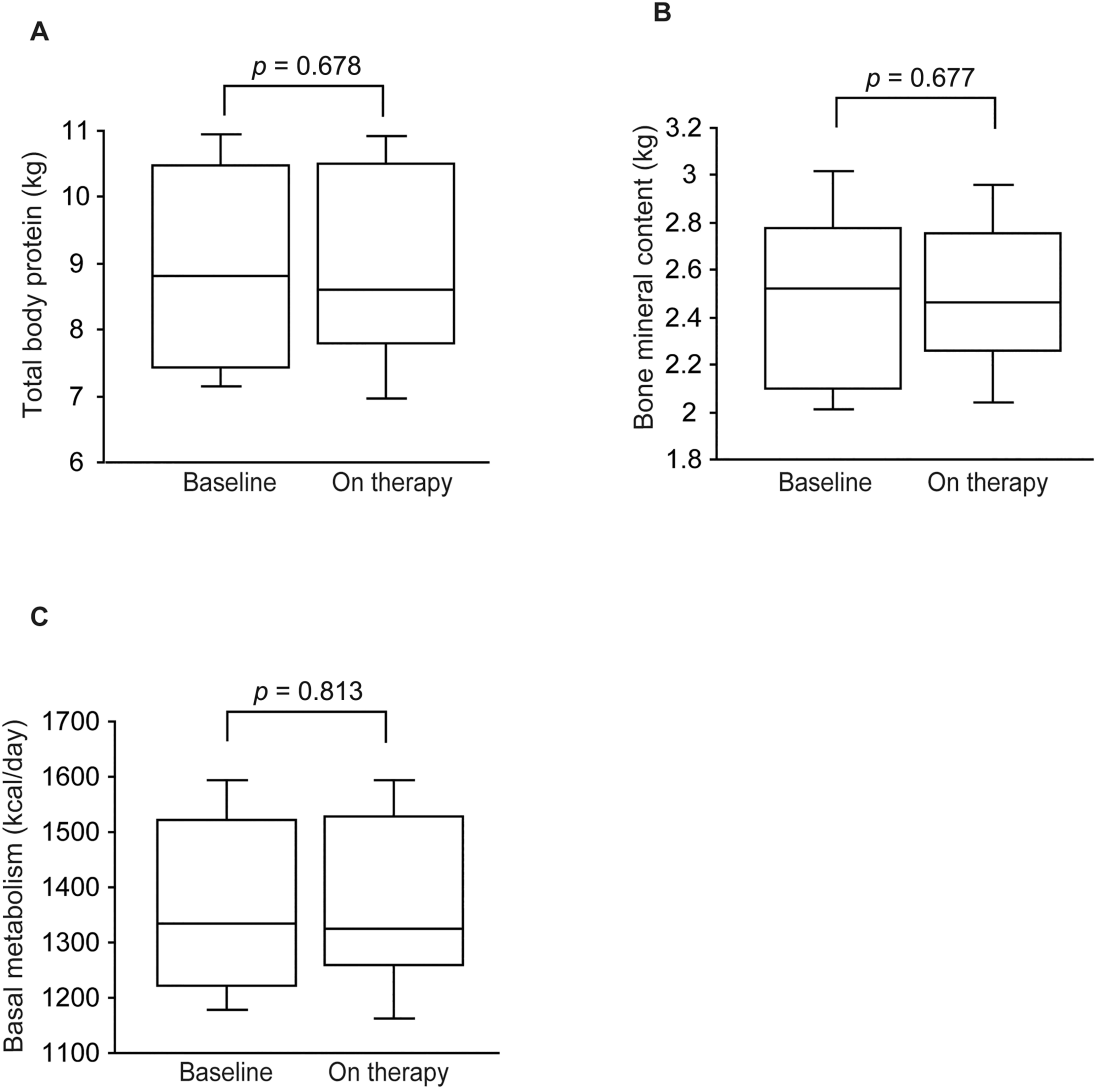
Box plots showing total body protein (kg) (A), bone mineral content (kg) (B) and basal metabolism (C) at baseline and after tirzepatide treatment. The duration of tirzepatide treatment was 4 (IQR 3–6) months.

Protein content, bone mineral content and basal metabolism remained unchanged during the observation period (8.9 ± 1.6 vs. 8.9 ± 1.5 kg, *p* = 0.678; 2.5 ± 0.4 vs. 2.5 ± 0.3 kg, *p* = 0.677; 1364 ± 167 vs. 1372 ± 165 kcal, *p* = 0.813, respectively) (Figure 6A–C). Lastly, changes in DW and body composition were examined in six patients who switched from dulaglutide to tirzepatide, DW and body fat were significantly decreased (*p* = 0.028, respectively) (Table 2).

**TABLE 2:** Changes in body composition after switching from dulaglutide to tirzepatide.

	Under treatment with dulaglutide	After change of tirzepatide	*p* Value
DW (mean ± SD; kg)	75.5 ± 11.7	72.9 ± 13.3	0.028
ICW (mean ± SD; %)	20.3 ± 3.7	20.7 ± 3.5	0.167
ECW (mean ± SD; %)	13.5 ± 2.2	13.9 ± 2.3	0.115
Body fat (mean ± SD; %)	39.1 ± 10.7	35.6 ± 10.1	0.028
Muscle (mean ± SD; %)	43.1 ± 7.5	44.2 ± 7.3	0.115

Abbreviations: DW, dry weight; ECW, extracellular water; ICW, intracellular water; SD, standard deviation.

## 4 Discussion

Our findings revealed that the reduction in DW observed during tirzepatide treatment stemmed from changes in volume status, including reductions in ICW, ECW and TBW and a decrease in body fat content. The reduction in DW because of tirzepatide treatment was −3.5 ± 0.5 kg and was also observed over an extended period of time; however, the effect was observed mainly in body fat content, showing no discernible impact on skeletal muscle mass and SMI. Recent studies have demonstrated that a decrease in SMI, or the appearance of sarcopenia and frailty, is often observed in patients with CKD, which shortens their life expectancy [13]. In this regard, tirzepatide only reduces fat and does not affect muscle mass, thereby eliminating concerns regarding sarcopenia and frailty in patients with DKD undergoing HD.

PEW, the wasting of muscle and fat, is prevalent in patients undergoing HD and is strongly linked to mortality; the ECW/TBW ratio has been proposed as a possible marker for protein‐energy wasting and mortality, as a ratio exceeding 0.4 correlates with an elevated risk of mortality associated with protein‐energy wasting [13]. In our study, the ECW/TBW ratio with tirzepatide administration was 0.3, suggesting a low risk of protein‐energy wasting or frailty [14]. However, the large fluctuations in TBW in HD patients may be a limitation of applying the BIA to HD patients.

Previous report showed that exposure to tirzepatide was similar in CKD and healthy participants: the 90% confidence intervals for the ratios of area under the plasma concentration–time curves (AUCs) and maximum plasma drug concentration comparing each renal impairment group to the normal renal function group were consistent, except for a 25%–29% increase in AUCs in the moderate renal impairment group [14]. Few adverse events were reported across the renal impairment group in this study, which are similar to our results.

The effect of GIP on the kidney is not clear, whereas the effect of GLP‐1 on the kidneys, for which the GLP‐1 receptor is mainly found in the glomeruli, has been shown by our group [15]. Interestingly, GLP‐1 receptor is decreased in the renal cortex of patients with long‐term Type 1 diabetes (The Joslin 50‐year medallist study at Joslin Diabetes Center, Harvard University; Mima A. and King G.L., unpublished observation). We have also demonstrated that GLP‐1 receptor agonists could increase the cyclic adenosine monophosphate/protein kinase A pathway and decrease extracellular signal‐regulated kinase1/2/plasminogen activator inhibitor‐1 pathway, which is activated by protein kinase Cβ/angiotensin II. Furthermore, GLP‐1 receptor agonist decreased inflammatory cytokines that can play a significant role in developing DKD [15].

Like our results, a previous study using semaglutide in HD patients showed a weight loss of about 10% [16].

Switching from insulin to liraglutide was effective in HD patients with T2DM, especially in those who had difficulty controlling blood water content because of failure of dietary restriction, resulting especially in a decrease in CTR [17].

However, there is no report that has clarified the effect of GLP‐1 receptor agonist on CTR. In our study, tirzepatide did not reduce muscle mass; in fact, tirzepatide has been shown to significantly decrease fat mass in Japanese patients with T2DM with little change in muscle mass [18]. It has recently been reported that the inhibition of glucagon action in mice increases muscle mass, suggesting that the mechanism by which tirzepatide protects muscle mass may include postprandial glucagon [19]. Since glucagon changes significantly before and after HD, it is possible that the effect of glucagon on muscle mass may be significant in HD patients [20]. In other words, the effect of tirzepatide on glucagon may be important in terms of muscle mass retention in HD patients.

## 5 Conclusion

The effect of tirzepatide on TBW reductions in patients with T2DM undergoing HD was large, resulting in lower DW. This weight loss was primarily due to fat loss and partially due to a decrease in extracellular fluid; however, tirzepatide did not reduce muscle mass. The ability to lower DW may result in less ultrafiltration rate. During the observation period, no adverse events except nausea were observed. Furthermore, we have demonstrated that the administration of tirzepatide could be useful to improve the prognosis of patients with T2DM undergoing HD.

## Author Contributions

A.M. contributed to the conception and design of the study and wrote the first draft of the manuscript. A.M. and Y.H. prepared the material, collected the data and involved in the analyses.

## Conflicts of Interest

A.M. received speaker's honorarium from Kyowa Kirin, Sumitomo Pharma, Torii, Bayer, Eli Lilly, Mochida and Boehringer Ingelheim. A.M. has also received research grants from Kyowa Kirin, Sumitomo Pharma, Chugai, Mochida, Torii and Boehringer Ingelheim.

References1
International Diabetes Federation
, 2021, http://www.idf.org/.2
Centers for Disease Control and Prevention
, 2020, http://www.cdc.gov.3
Collaboration GBDCKD
, “Global, Regional, and National Burden of Chronic Kidney Disease, 1990–2017: A Systematic Analysis for the Global Burden of Disease Study 2017,” Lancet
395, no. 10225 (2020): 709–733.32061315
10.1016/S0140-6736(20)30045-3PMC70499054
Diabetes Control and Complications Trial Research Group
, “The Effect of Intensive Treatment of Diabetes on the Development and Progression of Long‐Term Complications in Insulin‐Dependent Diabetes Mellitus,” The New England Journal of Medicine
329, no. 14 (1993): 977–986.8366922
10.1056/NEJM1993093032914015

C. J.
Bailey
 and 
P. J.
Grant
, “The UK Prospective Diabetes Study,” Lancet
352, no. 9144 (1998): 1932; author reply 1934.10.1016/S0140-6736(98)00090-798638066

A.
Mima
, 
T.
Matsubara
, 
H.
Arai
, et al., “Angiotensin II‐Dependent Src and Smad1 Signaling Pathway Is Crucial for the Development of Diabetic Nephropathy,” Laboratory Investigation
86, no. 9 (2006): 927–939.16767106
10.1038/labinvest.37004457

D. J.
Drucker
, “The Biology of Incretin Hormones,” Cell Metabolism
3, no. 3 (2006): 153–165.16517403
10.1016/j.cmet.2006.01.0048

A.
Mima
, “A Narrative Review of Diabetic Kidney Disease: Previous and Current Evidence‐Based Therapeutic Approaches,” Advances in Therapy
39, no. 8 (2022): 3488–3500.35751762
10.1007/s12325-022-02223-09

A.
Mima
, 
A.
Nomura
, and 
T.
Fujii
, “Current Findings on the Efficacy of Incretin‐Based Drugs for Diabetic Kidney Disease: A Narrative Review,” Biomedicine & Pharmacotherapy
165 (2023): 115032.37331253
10.1016/j.biopha.2023.11503210

A.
Mima
, 
H.
Gotoda
, 
R.
Lee
, 
A.
Murakami
, 
R.
Akai
, and 
S.
Lee
, “Effects of Incretin‐Based Therapeutic Agents Including Tirzepatide on Renal Outcomes in Patients With Type 2 Diabetes: A Systemic Review and Meta‐Analysis,” Metabol Open
17 (2023): 100236.36923991
10.1016/j.metop.2023.100236PMC1000929311

T.
Yajima
 and 
K.
Yajima
, “Ratio of Extracellular Water to Intracellular Water and Simplified Creatinine Index as Predictors of All‐Cause Mortality for Patients Receiving Hemodialysis,” PLoS One
18, no. 3 (2023): e0282864.36897875
10.1371/journal.pone.0282864PMC1000456312

E.
Nagy
, 
E.
Samaan
, 
M.
El‐Gamal
, 
M.
Shamsuddin
, and 
S.
Tharwat
, “Concordance Between Muscle Mass Assessed by Bioelectrical Impedance Analysis and by Muscle Ultrasound: A Cross‐Sectional Study in a Cohort of Patients on Chronic Hemodialysis,” BMC Nephrology
25, no. 1 (2024): 49.38321408
10.1186/s12882-024-03487-0PMC1084838213

T.
Yajima
 and 
K.
Yajima
, “Association of Extracellular Water/Total Body Water Ratio With Protein‐Energy Wasting and Mortality in Patients on Hemodialysis,” Scientific Reports
13, no. 1 (2023): 14257.37652929
10.1038/s41598-023-41131-3PMC1047167614

S.
Urva
, 
T.
Quinlan
, 
J.
Landry
, 
J.
Martin
, and 
C.
Loghin
, “Effects of Renal Impairment on the Pharmacokinetics of the Dual GIP and GLP‐1 Receptor Agonist Tirzepatide,” Clinical Pharmacokinetics
60, no. 8 (2021): 1049–1059.33778934
10.1007/s40262-021-01012-2PMC833259615

A.
Mima
, 
J.
Hiraoka‐Yamomoto
, 
Q.
Li
, et al., “Protective Effects of GLP‐1 on Glomerular Endothelium and Its Inhibition by PKCbeta Activation in Diabetes,” Diabetes
61, no. 11 (2012): 2967–2979.22826029
10.2337/db11-1824PMC347851816

J. C.
De la Flor
, 
J. D.
Lorenzo
, 
A.
Marschall
, 
F.
Valga
, 
T. M.
Vazquez
, and 
E. R.
Cicero
, “Efficacy and Safety of Semaglutide, a Glucagon‐Like Peptide‐1 Receptor Agonist in Real‐Life: A Case Series of Patients in Maintenance Incremental Hemodialysis,” Case Reports in Nephrology and Dialysis
12, no. 3 (2022): 238–247.36465574
10.1159/000527919PMC971044117

M.
Kondo
, 
M.
Toyoda
, 
M.
Kimura
, 
N.
Ishida
, and 
M.
Fukunaga
, “Favorable Effect on Blood Volume Control in Hemodialysis Patients With Type 2 Diabetes After Switching From Insulin Therapy to Liraglutide, a Human Glucagon‐Like Peptide‐1 Analog—Results From a Pilot Study in Japan,” The Tokai Journal of Experimental and Clinical Medicine
42, no. 1 (2017): 52–57.28413872
18

D.
Yabe
, 
D.
Kawamori
, 
Y.
Seino
, 
T.
Oura
, and 
M.
Takeuchi
, “Change in Pharmacodynamic Variables Following Once‐Weekly Tirzepatide Treatment Versus Dulaglutide in Japanese Patients With Type 2 Diabetes (SURPASS J‐Mono Substudy),” Diabetes, Obesity & Metabolism
25, no. 2 (2023): 398–406.10.1111/dom.14882PMC100921543618478019

S.
Ueno
, 
Y.
Seino
, 
S.
Hidaka
, et al., “Blockade of Glucagon Increases Muscle Mass and Alters Fiber Type Composition in Mice Deficient in Proglucagon‐Derived Peptides,” Journal of Diabetes Investigation
14, no. 9 (2023): 1045–1055.37300240
10.1111/jdi.14032PMC1044520020

Y.
Yoshizawa
, 
M.
Hosojima
, 
H.
Kabasawa
, et al., “Measurement of Plasma Glucagon Levels Using Mass Spectrometry in Patients with Type 2 Diabetes on Maintenance Hemodialysis,” Kidney & Blood Pressure Research
46, no. 5 (2021): 652–656.34515141
10.1159/000518027

## Data Availability

The data that support the findings of this study are available in the figshare at https://figshare.com/account/items/25211795/edit, but restrictions apply to the availability of these data, and so are not publicity available. Data are, however, available from the corresponding author upon reasonable request and with permission of figshare.
